# High-Dose Caffeine Mouth Rinse Is an Effective Strategy for Isokinetic Strength Generation in Soccer Players

**DOI:** 10.5114/jhk/208389

**Published:** 2025-11-19

**Authors:** Mehmet Vural, Mustafa Ozdal, Cemre Didem Eyipınar, Mete Berk Demiryol

**Affiliations:** 1 Coaching Education Department, Faculty of Sport Science, Gaziantep University, Gaziantep, Turkey.; 2 Physical Education and Sports Teaching Department, Faculty of Sport Science, Gaziantep University, Gaziantep, Turkey.; 3 Sport Management Department, Faculty of Sport Science, Gaziantep University, Gaziantep, Turkey.

**Keywords:** performance, ergogenic aids, anhydrous caffeine, supplementation

## Abstract

Caffeine supplementation is one of the most commonly used ergogenic aids among soccer players. However, research shows conflicting results regarding the effects of different caffeine forms on muscle strength and power. This study investigated the impact of a high-dose caffeine mouth rinse on isokinetic knee extensor and flexor strength of the dominant leg at high (180°/s^−1^) and low (60°/s^−1^) angular velocities. Eight young male soccer players were randomly assigned to three conditions. Ten seconds before the isokinetic strength tests, participants either rinsed with an aspartame solution containing 750 mg of caffeine (Experimental), a calorie-free aspartame solution (Placebo) or did not use any solution (Control). Participants continued the mouth rinse for 10 s. Isokinetic strength measurements were performed at angular velocities of 60°/s^−1^ (5 repetitions) and 180°/s^−1^ (15 repetitions) in all interventions. Peak torque (Nm), the H/Q ratio, time to peak torque (ms), and average peak torque (Nm) in extension and flexion were analyzed. Data were tested for normality using the Shapiro-Wilk test, followed by one-way ANOVA and LSD post hoc tests (significance set at p < 0.05). According to the results, a significant difference was found in the H/Q ratio and time to peak torque at the angular velocity of 60°/s^−1^ (p < 0.05, η^2^ = 0.290; 0.306). At the angular velocity of 180°/s^−1^, no significant differences were found among the interventions (p > 0.05). These findings suggest that a high dose of the caffeine mouth rinse was effective at the lower angular velocity (60°/s^−1^), but had no effect on peak torque at the higher angular velocity (180°/s^−1^).

## Introduction

Caffeine is an alkaloid derived from methylxanthines and is one of the most widely used psychoactive drugs globally. It is well known for its ergogenic properties which help athletes perform better (Baltazar-Martins et al., 2020; Giráldez-Costas et al., 2023; Guest et al., 2021). Despite its performance-enhancing effects, some individuals may experience adverse reactions, such as nervousness, headaches, and high blood pressure, after consuming caffeine. Therefore, the caffeine mouth rinse has been studied as an alternative and effective method that bypasses gastrointestinal absorption and metabolism. In caffeine mouth rinsing, caffeine is swished in the mouth for 5 to 20 s without swallowing (Karayigit et al., 2021; Pallares et al., 2013; Van Cutsem et al., 2018). The ergogenic effects of the caffeine mouth rinse remain debatable and require further investigation (Ehlert et al., 2020).

Research has shown that the potential performance-enhancing effects of caffeine mouth rinsing may occur through several mechanisms. The primary mechanism involves caffeine binding to adenosine receptors in the buccal mucosa, which promotes an increase in the release of neurotransmitters. The secondary mechanism involves caffeine interacting with bitter-taste receptors in the oral cavity. These receptors may stimulate nerves connected to the cerebral cortex (da Silva et al., 2023). According to the literature, several studies have found that bitter-tasting substances can improve performance by signaling to the brain that the body is ready to take action. Furthermore, buccal mucosa-mediated absorption of caffeine has led researchers to examine the effect of caffeine mouth rinsing on athletic performance (Pickering, 2019).

In soccer, lower-body muscle power and strength are essential for athletic performance. Isokinetic strength tests have recently gained importance in evaluating athletic capacity and determining performance requirements. The strength output obtained through isokinetic contractions can be interpreted as the maximal force that muscles can produce (Impellizzeri et al., 2008). The knee joint is the most commonly tested site for isokinetic strength evaluations. In addition to the force generated at this joint, the ratio between the opposing muscles, hamstrings, and quadriceps, is also significant for sports. This ratio provides insight into muscle balance, which is necessary for effective performance and reduced injury risk. Furthermore, the maximal strength values produced by the muscles offer valuable information about the athlete’s suitability for physical loading and their potential to reach optimal performance levels.

In summary, studies on isokinetic strength and related variables suggest that isokinetic measurements provide important information about athletes' injury risk, readiness levels, and muscle balance. Such measurements have been examined across various sports, age groups, athletes categories and competitive levels (Cheung et al., 2012; França et al., 2024 Grygorowicz et al., 2010; Kim and Hong, 2011). In addition to evaluating knee isokinetic strength, some studies have investigated the mechanisms influencing improvements in isokinetic test results. Various warm-up types have been explored in this context, and multiple methods have been developed to improve isokinetic strength and muscle balance (Ferreira-Júnior et al., 2013).

Several randomized, double-blind, placebo-controlled crossover studies have also examined the effects of caffeine consumption and acute caffeine supplementation on isokinetic performance over the years (Ali et al., 2016; Bazzucchi et al., 2011; Grgic et al., 2022; Timmins and Saunders, 2014). In light of this evidence, it can be stated that strength output obtained from isokinetic tests is critical and that mechanisms that may enhance isokinetic strength are of particular interest, as these values are directly linked to field performance.

Recent research has demonstrated that the administration of placebos in experimental studies can also elicit an ergogenic effect (Grgic et al., 2020b; 2021). When examining current randomized, double- or single-blind, placebo-controlled crossover studies on the caffeine mouth rinse, it is evident that comparisons are generally made between experimental and placebo groups. However, the limited number of studies including a control group is noteworthy. This aspect has not been adequately examined in the context of alternative forms of caffeine supplementation and their effects on isokinetic strength (Barbosa et al., 2020; de Albuquerque Melo et al., 2021; Marinho et al., 2024; Nabuco et al., 2021). This is important, as mouth rinsing minimizes the potential side effects of caffeine (since it is not swallowed) and allows for rapid action via the buccal mucosa.

According to the occupancy hypothesis, higher doses of caffeine excite a greater number of adenosine and bitter taste receptors in the buccal mucosa, leading to improved muscular performance (Clark, 1926). In line with this hypothesis, the specific effect of a high dose of caffeine mouth rinse on isokinetic strength remains unknown, indicating a need for further investigation into its efficacy compared to other caffeine delivery methods (Ehlert et al., 2020). Additionally, no study to date has analyzed the effects of an alternative form of caffeine (e.g., mouth rinsing) on isokinetic strength at both low and high angular velocities.

Our study aimed to address this gap in the literature and provide preliminary findings for future research. In this context, we investigated the effects of high doses of the caffeine mouth rinse on the isokinetic strength of knee extensors and flexors of the dominant leg at high (180°/s^−1^) and low (60°/s^−1^) angular velocities in soccer players. We hypothesized that this alternative form of caffeine supplementation (mouth rinsing) might improve isokinetic muscle strength performance, which is particularly important in soccer. Moreover, it was hypothesized that caffeine mouth rinses at the high angular velocity (180°/s^−1^) will enhance rapid strength performance, which is critical for actions such as acceleration, changing direction, shooting, and pressing in soccer, whereas supplementation at the low angular velocity (60°/s^−1^) would improve maximal strength, important for jumping, passing, shooting, and short-distance sprints.

## Methods

### Participants

The number of participants in this study was determined based on a previous study (Grgic et al., 2022), which examined whether caffeine had a significant effect on isokinetic strength, power, and endurance. That study reported an effect size of 0.7708429 for peak torque in isokinetic knee extension at 60°/s^−1^, and as a result of statistical power analysis using G*Power 3 software, it was determined that at least seven participants were needed for this study, with an alpha value of 0.05 and a power value of 0.95. To ensure an adequate sample size, eight participants were selected for this study. Based on this analysis, eight amateur soccer players volunteered (age: 20 ± 1 years; body height: 180.5 ± 6.785 cm; body mass: 72.75 ± 7.92 kg), each with approximately seven years of soccer training experience and a minimum of three exercise sessions per week. The anthropometric characteristics of participants, including body mass, body height, and age, were thoroughly documented to provide the essential context for this study (Table 1).

**Table 1:** Descriptive statistics of demographic characteristics of the participants.

	N	Minimum	Maximum	Mean (X̄)	Std. Deviation (SD)
Age (year)	8	19	22	20.50	1.195
Body mass (kg)		60	85	72.75	7.924
Body height (cm)		168	187	180.5	6.845

Note: kg: kilogram; cm: centimeter

To mitigate the potential confounding effects of caffeine consumption, participants were provided with detailed information regarding common food and beverage products that contain caffeine and were instructed to refrain from consuming these products throughout the study period. Furthermore, participants abstained from supplements, medications, tobacco, alcohol, and caffeinated beverages prior to the commencement of data collection and throughout the experimental period.

To minimize variability in measurements due to individual differences, the eight volunteers were randomly assigned to different interventions on separate days, with measurements conducted accordingly. The angular velocity at which isokinetic measurements would be performed, along with the specific application, was determined on the test day. All participants were given a clear explanation of the study, including the potential risks and benefits of participation, and signed an informed consent form prior to participation. Ethical approval for the study was obtained from the Gaziantep University Social and Human Sciences Ethics Committee, Gaziantep, Turkey (approval code: 347008/28, approval date: 06 July 2023), and the study was conducted following the Helsinki Declaration.

## Measures

### Mouth Rinse Protocol

Immediately before the test began, participants were given a 25-mL mouth rinse solution in a transparent plastic cup and asked to swish the solution around in their mouths for 10 s. A 10-s rinse has been shown not to raise blood caffeine levels (Doering et al., 2014). The experimental group used 25 mL of a 3% caffeine solution containing 750 mg of anhydrous caffeine (Nature’s Supreme Caffeine, TR) and 18 mg of aspartame (Canderel, Merisant, TR). For the placebo rinse, distilled water was used with 18 mg of the non-caloric artificial sweetener aspartame. The control group did not rinse their mouths. Aspartame was included in all solutions to ensure similarity in taste and appearance.

**Figure 1:**
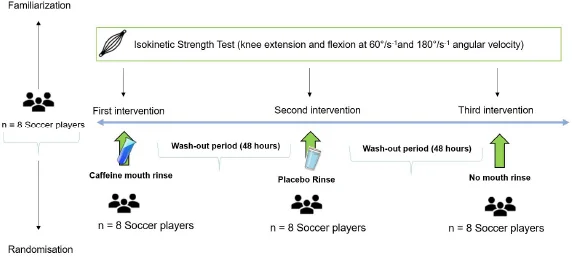
Schematic representation of the experimental process.

The caffeine dosage of 750 mg was selected because it has been demonstrated to be effective for muscular strength (Karayigit et al., 2021). All solutions were administered immediately before the trials. Before each trial, participants refrained from caffeine intake and strenuous exercise for 48 hours. A two-day washout period was implemented between each intervention (Figure 1).

### Isokinetic Measurement Protocol

Isokinetic strength testing was performed using a CSMI-brand isokinetic device (Humac Norm Testing and Rehabilitation System, CSMI, USA) at the Performance Measurement and Physiology Laboratory, Faculty of Sports Sciences, Gaziantep University. The device recorded measurements on a connected computer. Although there are several different methods for measuring the variables required in studies, it is beneficial to use measurement methods that have been tested for validity and reliability. Therefore, it is advisable to choose the test that best suits the biomechanical properties necessary for achieving functional goals or success (Juan-Recio et al., 2024). The system used in this study has demonstrated validity and reliability (ICC: 0.74 to 0.89) at angular velocities of 60°/s^−1^ and 180°/s^−1^ during knee extension and flexion (Habets et al., 2018).

Before the primary test measurements, participants performed 10 warm-up repetitions at 300°/s^−1^ to familiarize themselves with the device and the resistance. Participants were positioned according to standard isokinetic measurement protocols. The dynamometer and supporting apparatus were adjusted to match the specified angles. Knee joint motion angles were set to 90° in flexion and 0° in extension. The arm axis of the dynamometer was aligned parallel to the subject's leg, with the pivot point placed at the lateral condyle of the femur. Adjustments were made for individual physical differences. Exercises were performed at all angular velocities based on these settings. The participants' bodies and measured extremities were secured to the chair with straps that did not interfere with muscle output. Two mechanical stoppers at the flexion and extension points ensured the joint remained within the predetermined range of motion (Cabri, 1991).

Isokinetic strength measurements were conducted at two different angular velocities: 60°/s^−1^ (5 repetitions) and 180°/s^−1^ (15 repetitions), with rest periods of 30 s between sets and 60 s between angular velocities. The recorded values included the H/Q ratio (%), peak torque (PT, Nm), average peak torque (AP, Nm), and time to peak torque (ms).

### Design and Procedures

This study was designed as a randomized, controlled, double-blind crossover experiment (Figure 1). Randomization was performed using a complete randomization method. Participants were asked to draw colored cards, each representing a different intervention. Upon arriving at the lab, each participant randomly selected a card without knowing which treatment it represented and completed the corresponding application that day.

The study was double-blinded: participants were unaware of which supplement they received, and a separate researcher was also blind to the treatment assignments. A two-day washout period was employed between interventions. This washout period allowed for the clearance of the active ingredient from the body before initiating a new trial.

As illustrated in Figure 1, the study was completed over one week. Participants visited the laboratory on three occasions. During the first visit, age, body height, and body mass were recorded. The intervention and isokinetic measurement for each day were determined by randomly drawn cards, and the isokinetic test was performed immediately afterward. The three treatments were: a caffeine mouth rinse, a placebo rinse, and a control condition (no rinse), all randomized in order. Isokinetic tests were conducted at two angular velocities: 60°/s^−1^ (5 repetitions) and 180°/s^−1^ (15 repetitions).

### Statistical Analysis

Statistical analysis was performed using SPSS version 22.0 (SPSS Inc., Chicago, IL, USA). Values were presented as minimum, maximum, mean, and standard deviation. The significance level was set at 0.05. The Shapiro-Wilk test was used to assess normality. One-way ANOVA and LSD post hoc tests were conducted to evaluate differences between trials. Effect sizes were analyzed using the partial eta-squared (η^2^) statistic. According to this classification, effect sizes were considered non-significant (< 0.10), moderate (0.25–0.39), or large (≥ 0.40).

## Results

Data were collected from eight participants who completed all three treatments on separate days. These data were analyzed using a statistical software program. Based on the isokinetic measurement device output, the following variables were obtained: the hamstring-to-quadriceps (H/Q) ratio, extension peak torque, flexion peak torque, extension average peak torque, flexion average peak torque, and time to peak torque, each measured at angular velocities of 60°/s^−1^ and 180°/s^−1^ during both extension and flexion movements.

After confirming normality using the Shapiro-Wilk test, a one-way ANOVA was applied to compare group differences. When significant differences were found, the LSD post hoc test was used to determine which treatment conditions differed. Effect sizes were calculated using eta squared (η^2^) to indicate the magnitude of significant effects.

Table 2 presents the statistical analysis results comparing the three treatments at both 60°/s^−1^ and 180°/s^−1^ angular velocities.

**Table 2:** Comparison of isokinetic strength averages obtained at 60°/s^−1^ and 180°/s^−1^ angular velocities between interventions.

			Mean	Std. D.	Std. E.	F	*p*	η2
**180°/s^−1^**	H/Q(%)	Con.	62.00	10.13	3.58	3.074	0.067	0.234
		Exp.	50.25	7.76	2.74			
		Plc.	60.63	12.63	4.46			
	Pt_ex_ (Nm)	Con.	144.00	26.20	9.26	0.365	0.698	0.034
		Exp.	156.13	31.07	10.98			
		Plc.	149.75	27.67	9.78			
	AP_ex_(Nm)	Con.	254.75	54.40	19.23	0.118	0.889	0.011
		Exp.	267.88	58.07	20.53			
		Plc.	266.25	63.89	22.59			
	Time to peak torque_ex_ (Ms)	Con.	0.24	0.03	0.01	0.813	0.457	0.066
		Exp.	0.26	0.04	0.01			
		Plc.	0.25	0.04	0.01			
	Pt_flx_ (Nm)	Con.	89.25	22.07	7.80	0.444	0.648	0.040
		Exp.	79.88	25.65	9.07			
		Plc.	90.13	24.53	8.67			
	AP_flx_ (Nm)	Con.	159.50	46.59	16.47	0.237	0.791	0.022
		Exp.	144.00	57.40	20.29			
		Plc.	161.25	60.63	21.44			
	Time to peak torque_flx_ (Ms)	Con.	0.27	0.04	0.02	0.270	0.766	0.031
		Exp.	0.27	0.03	0.01			
		Plc.	0.26	0.04	0.01			
**60°/s^−1^**	H/Q (%)	Con.	62.00**^a^**	8.43	2.98	4.292	0.027*	0.290
		Exp.	51.13	10.30	3.64			
		Plc.	63.38**^a^**	8.63	3.05			
	Pt_ex_ (Nm)	Con.	221.75	39.60	14.00	1.257	0.305	0.010
		Exp.	248.25	38.44	13.59			
		Plc.	219.75	42.25	14.94			
	AP_ex_ (Nm)	Con.	155.13	31.62	11.18	1.410	0.266	0.013
		Exp.	176.75	28.84	10.20			
		Plc.	155.75	27.32	9.66			
	Time to peak torque_ex_ (Ms)	Con.	0.48	0.08	0.03	4.682	0.021*	0.306
		Exp.	0.58	0.04	0.01			
		Plc.	0.48	0.10	0.03			
	Pt_flx_ (Nm)	Con.	135.25	17.41	6.15	0.707	0.504	0.063
		Exp.	125.75	25.82	9.13			
		Plc.	137.00	16.55	5.85			
	AP_flx_ (Nm)	Con.	109.00	17.07	6.04	1.205	0.319	0.012
		Exp.	97.13	19.05	6.74			
		Plc.	107.88	14.19	5.02			
	Time to peak torque_flx_ (Ms)	Con.	0.51	0.14	0.05	0.517	0.604	0.046
		Exp.	0.48	0.14	0.05			
		Plc.	0.44	0.09	0.03			

Note: *: Significant difference between groups (p < 0.05); H/Q: Hamstring/Quadriceps; Pt_ex_: Extension Peak Torque; Pt_flx:_ Flexion Peak Torque; AP_ex:_ Extension Average Peak Torque; AP_flx:_ Flexion Average Peak Torque; Ex: Extension; Flx: Flexion; Nm: Newtonmeter; Ms: Millisecond; a: Significant difference compared to the experimental group; Con: Control group; Exp: Experimental group (Caffeine Mouth rinse); Plc: Placebo Rinse Group

At 60°/s^−1^, a statistically significant difference was observed between treatments for the H/Q ratio, favoring the experimental group (*p* < 0.05; η^2^ = 0.290), indicating a moderate effect size. Additionally, time to peak torque at this velocity showed a significant difference with a moderate effect (*p* < 0.05; η^2^ = 0.306), again favoring the experimental condition.

However, at 180°/s^−1^, no significant differences were found among treatments for any of the following variables: the H/Q ratio, extension peak torque, flexion peak torque, extension average peak torque, flexion average peak torque, or time to peak torque (*p* > 0.05). Similarly, at 60°/s^−1^, no significant treatment differences were found for extension peak torque, flexion peak torque, extension average peak torque, or flexion average peak torque (*p* > 0.05).

**Figure 2:**
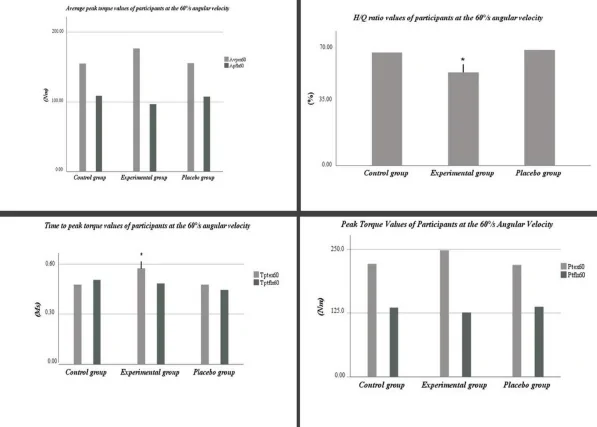
Comparison of participants' isokinetic variables at the 60°/s^−1^ angular velocity among interventions. Ms: Millisecond; Nm: Newtonmeter; Ex: Extension; Flx: Flexion; %: Percent; Avpex60: Average peak torque extension at the 60°/s^−1^ angular velocity; Avpflx60: Average peak torque flexion at the 60°/s^−1^ angular velocity; Tptex60: Time to peak torque extension at the 60°/s^−1^ angular velocity; Tptflx60: Time to peak torque flexion at the 60°/s^−1^ angular velocity; Ptex60: Peak torque extension at the 60°/s^−1^ angular velocity; Ptflx60: Peak torque flexion at the 60°/s^−1^ angular velocity; *: significant difference compared to the experimental group (p < 0.05)

Figures 1 and 2 illustrate the group-wise comparisons and trends for all measured variables at both angular velocities, offering a clearer visual interpretation of the results.

**Figure 3:**
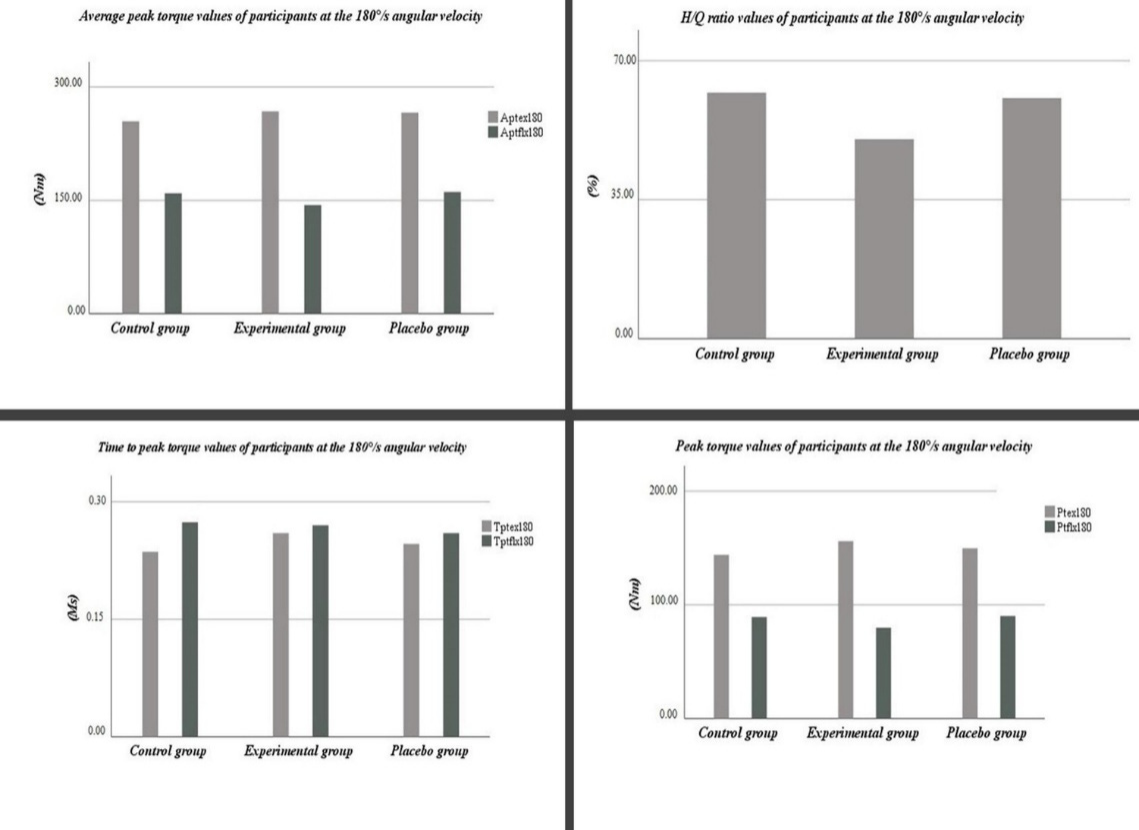
Comparison of participants' isokinetic variables at the 180°/s^−1^ angular velocity among interventions. Ms: Millisecond; Nm: Newtonmeter; Ex: Extension; Flx: Flexion; %: Percent; Avpex180: Average peak torque extension at the 180°/s^−1^ angular velocity; Avpflx180: Average peak torque flexion at the 180°/s^−1^ angular velocity; Tptex180: Time to peak torque extension at the 180°/s^−1^ angular velocity; Tptflx180: Time to peak torque flexion at the 180°/s^−1^ angular velocity; Ptex180: Peak torque extension at the 180°/s^−1^ angular velocity; Ptflx180: Peak torque flexion at the 180°/s^−1^ angular velocity

## Discussion

This study is the first to investigate the acute effects of high-dose caffeine mouth rinsing on knee isokinetic strength, offering an alternative to traditional caffeine supplementation methods that may have systemic side effects. While previous research has explored the impact of high-dose caffeine in ingestion or gum forms on muscular endurance and knee extensor/flexor strength, no prior study has specifically assessed the effects of caffeine mouth rinsing on isokinetic performance (Bond et al., 1986; Grgic and Pickering, 2019; Teimouri-Korani et al., 2025; Venier et al., 2019b). Thus, this study aimed to fill a critical gap by evaluating the impact of the caffeine mouth rinse on maximal muscle force during isokinetic contractions.

The results revealed significant differences in the H/Q ratio and time to peak torque at 60°/s^−1^, favoring the caffeine mouth rinse intervention. Time to peak torque is a critical metric that reflects how quickly peak force is generated and is associated with explosive strength and neuromuscular efficiency (Maciel et al., 2023). The observed improvements at this angular velocity suggest that caffeine mouth rinsing may enhance early-phase force production.

Prior studies have mostly focused on the acute or chronic effects of caffeine ingestion across various doses, with limited attention given to the mouth rinse form (Grgic et al., 2022). A meta-analysis has shown that caffeine ingestion can improve both upper and lower body strength and muscular endurance, with significant effects on lower limb strength at doses of 6 mg/kg or higher (Wu et al., 2024). Similarly, other trials have demonstrated improvements in psychomotor skills and muscular endurance following caffeine ingestion in athletic populations (Pereira et al., 2020; Santos et al., 2014).

However, the effects of caffeine on isokinetic performance remain mixed. Grgic and Pickering (2019) found in a meta-analysis that \caffeine had a small but significant effect on isokinetic peak torque, particularly in the knee extensors at higher angular velocities. Other studies have reported greater effects in eccentric versus concentric contractions (Wilk et al., 2019) and dose-dependent improvements in maximal force output in women (Filip-Stachnik et al., 2021).

Caffeine's ergogenic effects are thought to operate through several mechanisms. As an adenosine receptor antagonist, caffeine reduces perceived exertion and delays fatigue by increasing neurotransmitter release and motor unit recruitment (Cristina-Souza et al., 2022; Ryan et al., 2013). Moreover, recent findings suggest that caffeine may enhance neuromuscular activation and training efficiency, thereby contributing to improved performance outcomes during resistance exercise (Paoli et al., 2024). Caffeine also facilitates calcium ion mobilization from the sarcoplasmic reticulum, improving cross-bridge cycling and force production (Fryer and Neering, 1989; Tallis et al., 2012). Peripheral actions include enhancement of Na^+^/K^+^ pump function and increased calcium bioavailability, further supporting its role in muscle performance (Gonzalez et al., 2020).

Regarding the H/Q ratio, this metric is vital for assessing muscle balance. A balanced H/Q ratio (typically between 0.50 and 0.80) reflects coordinated function between agonist and antagonist muscle groups, reducing injury risk and enhancing performance (Maly et al., 2011). In this study, although extension peak torque was higher in the experimental group, the H/Q ratio appeared lower, likely due to greater caffeine-induced enhancement of quadriceps strength compared to hamstrings. This pattern may reflect training specificity, as participants (soccer players) are generally more accustomed to extension movements in traditional strength routines.

Similar trends have been noted in the literature. Warren et al. (2010) reported greater caffeine effects in the knee extensors than flexors. This may again be attributed to calcium-related mechanisms or habitual movement patterns favoring quadriceps activation. A meta-analysis in resistance-trained men also supported caffeine’s ergogenic effects, although H/Q ratios were not a direct focus (Grgic et al., 2018).

Despite these promising findings, no significant treatment differences were observed for extension/flexion peak torque or average peak torque at either 60°/s^−1^ or 180°/s^−1^ nor for the H/Q ratio and time to peak torque at 180°/s^−1^. These null findings align with previous research that has shown inconsistent responses to caffeine ingestion, particularly when using different doses or delivery methods. For instance, Venier et al. (2019a) reported improved peak and average torque with 300 mg caffeine gel, while Astorino et al. (2010) found that a 5 mg/kg dose enhanced peak knee flexion torque, but a 2 mg/kg dose did not, highlighting the importance of the dose and the administration method.

These inconsistencies suggest that individual variability, including genetic factors (e.g., ADORA2A polymorphisms), caffeine tolerance, and taste sensitivity, may influence the ergogenic response to caffeine (da Silva et al., 2023; Grgic et al., 2020a). Further research is warranted to determine the optimal caffeine mouth rinse dose, the timing relative to exercise, and the influence of individual characteristics on performance outcomes.

While the current study had a small sample size, it provides valuable preliminary insight into the potential of the caffeine mouth rinse as a practical ergogenic aid in isokinetic strength performance.

## Conclusions

As a result of the study, it was observed that caffeine mouth rinsing caused significant differences in time to peak torque of the knee extensors and in the H/Q ratio at the angular velocity of 60°/s^−1^ in isokinetic variables, with these differences favoring the experimental group. Caffeine mouth rinsing did not affect extension and flexion peak torque or mean power at angular velocities of 180°/s^−1^ and 60°/s^−1^. Conducting the study with control, experimental, and placebo groups and examining the acute effects of mouth rinsing on isokinetic variables distinguished this study from others in the literature. It contributed to the field by approaching the topic from a specific perspective. Further studies should assess the effects of additional variables such as the ideal dose of caffeine, timing between the last meal and exercise, training level, and inter-individual variation in the ability to detect bitter taste on the effectiveness of the caffeine mouth rinse (da Silva et al., 2023). Alternatively, future research could investigate dose-response relationships or different forms of caffeine supplementation on isokinetic performance at various angular velocities (e.g., 30–45°/s^−1^ or 120°/s^−1^).

Additionally, variations among subjects in the muscle fiber type, motivation, and caffeine responses should be investigated. Moreover, recent research suggests that genetic polymorphisms in carriers of the ADORA2A gene C allele (CC or CT genotype) affect the caffeine response after exercise (Grgic et al., 2020a). This highlights the need for more extensive trials and a deeper understanding of caffeine’s appropriate consumption.

This study has several limitations. First, the sample size was small. Another limitation is that, due to economic constraints, we were unable to assess participants’ blood caffeine concentrations and genetic status. These factors may have influenced the findings, emphasizing the importance of improving blinding techniques in future research to enhance methodological accuracy and reliability. Finally, caffeine consumption habits were not evaluated in this study. However, several studies have shown no effect of habitual caffeine consumption on sporting performance (Clarke and Richardson, 2021; Dodd et al., 1991; Gonçalves et al., 2017; Tarnopolsky and Cupido, 2000).

Soccer players might consider using a high-dose caffeine mouth rinse during isokinetic training instead of caffeine ingestion, which requires about 60 minutes to metabolize and may have potential adverse effects.

## References

[ref1] Ali, A., O’Donnell, J., Foskett, A., & Rutherfurd-Markwick, K. (2016). The influence of caffeine ingestion on strength and power performance in female team-sport players. *Journal of the International Society of Sports Nutrition*, 13, 1–9. 10.1186/s12970-016-0157-4 27980499 PMC5139084

[ref2] Astorino, T. A., Terzi, M. N., Roberson, D. W., & Burnett, T. R. (2010). Effect of two doses of caffeine on muscular function during isokinetic exercise. *Medicine and Science in Sports and Exercise*, 42(12), 2205–2210. 10.1249/MSS.0b013e3181e3a11d 20421833

[ref3] Baltazar-Martins, J. G., de Souza, D. B., Aguilar, M., Grgic, J., & Del Coso, J. (2020). Infographic. The road to the ergogenic effect of caffeine on exercise performance. *British Journal of Sports Medicine*, 54(10), 618–619. 10.1136/bjsports-2019-101018 31278086

[ref4] Barbosa, T. N., Parreira, L. K., Mota, J. F., Kalman, D., Saunders, B., & Pimentel, G. D. (2020). Acute caffeine mouth rinse does not improve performance in recreationally trained runners: a pilot study. *Nutrire*, 45, 1–6. 10.1186/s41110-020-00121-5

[ref5] Bazzucchi, I., Felici, F., Montini, M., Figura, F., & Sacchetti, M. (2011). Caffeine improves neuromuscular function during maximal dynamic exercise. *Muscle & Nerve*, 43(6), 839–844. 10.1002/mus.21995 21488053

[ref6] Bond, V., Gresham, K., McRae, J., & Tearney, R. J. (1986). Caffeine ingestion and isokinetic strength. *British Journal of Sports Medicine*, 20(3), 135–137. 10.1136/bjsm.20.3.135 3779343 PMC1478352

[ref7] Cabri J. M. H. (1991). Isokinetic strength aspects in human joints and muscles. *Applied Ergonomics*, 22(5), 299–302. 10.1016/0003-6870(91)90384-T 15676826

[ref8] Cheung, R., Smith, A., & Wong, D. (2012). H:Q ratios and bilateral leg strength in college field and court sports players. *Journal of Human Kinetics*, 33, 63–71. doi:10.2478/v10078-012-0045-1 23487043 PMC3588678

[ref9] Clark A. J. (1926). The reaction between acetyl choline and muscle cells. *Journal of Physiology*, 61(4), 530–546. 10.1113/jphysiol.1926.sp002314 16993813 PMC1514867

[ref10] Clarke, N. D., & Richardson, D. L. (2021). Habitual Caffeine Consumption Does Not Affect the Ergogenicity of Coffee Ingestion During a 5 km Cycling Time Trial. *International Journal of Sport Nutrition and Exercise Metabolism*, 31(1), 13–20. 10.1123/ijsnem.2020-0204 33260141

[ref11] Cristina-Souza, G., Santos, P. S., Santos-Mariano, A. C., Coelho, D. B., Rodacki, A., DE-Oliveira, F. R., Bishop, D. J., Bertuzzi, R., & Lima-Silva, A. E. (2022). Caffeine Increases Endurance Performance via Changes in Neural and Muscular Determinants of Performance Fatigability. *Medicine and Science in Sports and Exercise*, 54(9), 1591–1603. 10.1249/MSS.0000000000002944 35969166

[ref12] da Silva, W. F., Lopes-Silva, J. P., Camati Felippe, L. J., Ferreira, G. A., Lima-Silva, A. E., & Silva-Cavalcante, M. D. (2023). Is caffeine mouth rinsing an effective strategy to improve physical and cognitive performance? A systematic review. *Critical Reviews in Food Science and Nutrition*, 63(3), 438–446. 10.1080/10408398.2021.1949576 34275371

[ref13] de Albuquerque Melo, A., Bastos-Silva, V. J., Moura, F. A., Bini, R. R., Lima-Silva, A. E., & de Araujo, G. G. (2021). Caffeine mouth rinse enhances performance, fatigue tolerance and reduces muscle activity during moderate-intensity cycling. *Biology of Sport* 38(4), 517–523. 10.5114/biolsport.2021.100147 34937960 PMC8670801

[ref14] Dodd, S. L., Brooks, E., Powers, S. K., & Tulley, R. (1991). The effects of caffeine on graded exercise performance in caffeine naive versus habituated subjects. *European Journal of Applied Physiology and Occupational Physiology*, 62(6), 424–429. 10.1007/BF00626615 1893906

[ref15] Doering, T. M., Fell, J. W., Leveritt, M. D., Desbrow, B., & Shing, C. M. (2014). The effect of a caffeinated mouth-rinse on endurance cycling time-trial performance. *International Journal of Sport Nutrition and Exercise Metabolism*, 24(1), 90–97. 10.1123/ijsnem.2013-0103 23980239

[ref16] Dvir, Z. (2004) *Isokinetics muscle testing, interpretation, and clinical applications* (*2^nd^*). Churchill Livingstone.

[ref17] Ehlert, A. M., Twiddy, H. M., & Wilson, P. B. (2020). The effects of caffeine mouth rinsing on exercise performance: A systematic review. *International Journal of Sport Nutrition and Exercise Metabolism*, 30(5), 362–373. 10.1123/ijsnem.2020-0083 32668407

[ref18] Ferreira-Júnior, J., Vieira, C., Soares, S., Magalhães, I., Rocha-Júnior, V., Vieira, A., & Bottaro, M. (2013). Effects of different isokinetic knee extension warm-up protocols on muscle performance. *Journal of Sports Medicine and Physical Fitness*, 53(1–3), 25–9.

[ref19] Filip-Stachnik, A., Wilk, M., Krzysztofik, M., Lulińska, E., Tufano, J. J., Zajac, A., ... & Del Coso, J. (2021). The effects of different doses of caffeine on maximal strength and strength-endurance in women habituated to caffeine. *Journal of the International Society of Sports Nutrition*, 18(1), 25. 10.1186/s12970-021-00421-9 33781269 PMC8008648

[ref20] França, C., Martins, F., Przednowek, K., Marques, A., Ihle, A., Sarmento, H., & Gouveia, É. R. (2024). Knee and Hip Muscle Strength of Male Soccer Players from Different Competitive Levels. *Journal of Human Kinetics*, 93, 17–27. 10.5114/jhk/185217 39132414 PMC11307174

[ref21] Fryer, M. W., & Neering, I. R. (1989). Actions of caffeine on fast-and slow-twitch muscles of the rat. *Journal of Physiology*, 416, 435–454. 10.1113/jphysiol.1989.sp017770 2607458 PMC1189224

[ref22] Giráldez-Costas, V., Del Coso, J., Mañas, A., & Salinero, J. J. (2023). The long way to establish the ergogenic effect of caffeine on strength performance: an overview review. *Nutrients*, 15(5), 1178. 10.3390/nu15051178 36904177 PMC10005568

[ref23] Gonçalves, L. S., Painelli, V. S., Yamaguchi, G., Oliveira, L. F., Saunders, B., da Silva, R. P., Maciel, E., Artioli, G. G., Roschel, H., & Gualano, B. (2017). Dispelling the myth that habitual caffeine consumption influences the performance response to acute caffeine supplementation. *Journal of Applied Physiology*, 123(1), 213–220. 10.1152/japplphysiol.00260.2017 28495846

[ref24] Gonzalez, A. M., Guimarães, V., Figueiredo, N., Queiroz, M., Gentil, P., Mota, J. F., & Pimentel, G. D. (2020). Acute caffeine mouth rinse does not change the hydration status following a 10 km run in recreationally trained runners. *BioMed Research International*, 6598753. 10.1155/2020/6598753 32596348 PMC7298264

[ref25] Grgic J, Pickering C. (2019). The effects of caffeine ingestion on isokinetic muscular strength: a meta-analysis. *Journal of Science Medicine in Sport 22*, 353–360. doi: 10.1016/j.jsams.2018.08.016 30217692

[ref26] Grgic, J., Pickering, C., & Bishop, D. J. (2020a). ADORA2A C Allele carriers exhibit ergogenic responses to caffeine supplementation. *Nutrients*, 12, 741. 10.3390/nu12030741 32168870 PMC7146260

[ref27] Grgic, J., Trexler, E. T., Lazinica, B., & Pedisic, Z. (2018). Effects of caffeine intake on muscle strength and power: a systematic review and meta-analysis. *Journal of the International Society of Sports Nutrition*, 15(1), 11. 10.1186/s12970-018-02160 29527137 PMC5839013

[ref28] Grgic, J., Venier, S., Schoenfeld, B. J., & Mikulic, P. (2020b). Caffeine Ingestion Enhances Repetition Velocity in Resistance Exercise: A Randomized, Crossover, Double-Blind Study Involving Control and Placebo Conditions. *Journal of Human Kinetics*, 74, 177–183. 10.2478/hukin-2020-0023 33312285 PMC7706645

[ref29] Grgic, J., Venier, S., & Mikulic, P. (2022). Examining the effects of caffeine on isokinetic strength, power, and endurance. *Journal of Functional Morphology and Kinesiology*, 7(4), 71. 10.3390/jfmk7040071 36278732 PMC9590023

[ref30] Grgic, J., Venier, S., Mikulic, P. (2021). Both caffeine and placebo improve vertical jump performance compared with a non-supplemented control condition. *International Journal of Sports Physiology and Performance*, 16, 448–451. 10.1123/ijspp.2019-1028 32707561

[ref31] Grygorowicz, M., Kubacki, J., Pilis, W., Gieremek, K., & Rzepka, R. (2010). Selected Isokinetic Tests in Knee Injury Prevention. *Biology of Sport*, 27(1), 47–51.

[ref32] Guest, N. S., VanDusseldorp, T. A., Nelson, M. T., Grgic, J., Schoenfeld, B. J., Jenkins, N. D., ... & Campbell, B. I. (2021). International society of sports nutrition position stand: caffeine and exercise performance. *Journal of the International Society of Sports Nutrition*, 18(1), 1. 10.1186/s12970-020-00383-4 33388079 PMC7777221

[ref33] Habets, B., Staal, J. B., Tijssen, M., & van Cingel, R. (2018). Intrarater reliability of the Humac NORM isokinetic dynamometer for strength measurements of the knee and shoulder muscles. *BMC Research Notes*, 11(1), 15. 10.1186/s13104-018-3128-9 29321059 PMC5764011

[ref34] Impellizzeri F. M., Bizzini, M., Rampinini, E., Cereda, F., & Maffiuletti, N. A., (2008). Reliability of isokinetic strength imbalance ratios measured using the Cybex NORM dynamometer. *Clinical Physiology and Functional Imaging*, 28(2), 113–119. 10.1111/j.1475-097X.2007.00786.x 18070123

[ref35] Juan-Recio, C., Prat-Luri, A., Barbado, D., Vera-Garcia, F. J., & Moreno-Pérez, V. (2024). Reliability of a Trunk Flexion and Extensor Muscle Strength Test with Hand-Held and Isokinetic Dynamometers in Female Athletes. *Journal of Human Kinetics*, 92, 43–52. 10.5114/jhk/172640 38736593 PMC11079922

[ref36] Karayigit, R., Koz, M., Sánchez-Gómez, A., Naderi, A., Yildirim, U. C., Domínguez, R., & Gur, F. (2021). High Dose of Caffeine Mouth Rinse Increases Resistance Training Performance in Men. *Nutrients*, 13(11), 3800. 10.3390/nu13113800 34836058 PMC8617760

[ref37] Kim, D., & Hong, J. (2011). Hamstring to quadriceps strength ratio and noncontact leg injuries: A prospective study during one season. *Isokinetics and Exercise Science*, 19(1), 1–6. Doi: 10.3233/IES-2011-0406

[ref38] Maciel, D. G., Dantas, G. A. F., Cerqueira, M. S., Barboza, J. A. M., Caldas, V. V. D. A., de Barros, A. C. M., ... & de Brito Vieira, W. H. (2023). Peak torque angle, acceleration time and time to peak torque as additional parameters extracted from isokinetic test in professional soccer players: a cross-sectional study. *Sports Biomechanics*, 22(9), 1108–1119. 10.1080/14763141.2020.1784260 32673150

[ref39] Maly, T., Zahalka, F., & Mala, L. (2011). Differences between isokinetic strength characteristics of more and less successful professional soccer teams. *Journal of Physical Education and Sport*, 11(3), 306.

[ref40] Marinho, A. H., da Silva, J. M., Brandão, V. F. D. N., Jatobá, S. G., Júnior, P. B., Ataide-Silva, T., ... & de Araujo, G. G. (2024). Caffeine Mouth Rinse Plus Ingestion Improves the 10-Km Time Trial Compared to Caffeine Mouth Rinse Alone. *Research Quarterly for Exercise and Sport*, 95(3), 617–624 10.1080/02701367.2023.2293121 38271741

[ref41] Melo, A. A., Bastos-Silva, V. J., Moura, F. A., Bini, R. R., Lima-Silva, A. E., & de Araujo, G. G. (2021). Caffeine mouth rinse enhances performance, fatigue tolerance and reduces muscle activity during moderate-intensity cycling. *Biology of Sport*, 38(4), 517–523. 10.5114/biolsport.2021.100147 34937960 PMC8670801

[ref42] Nabuco, L. L., Saunders, B., da Silva, R. A. S., Molina, G. E., & Reis, C. E. G. (2021). Caffeine mouth rinse does not improve time to exhaustion in male trained cyclists. *International Journal of Sport Nutrition and Exercise Metabolism*, 31(5), 412–419. 10.1123/ijsnem.2020-0360 34311440

[ref43] Pallares, J. G., Fernandez-Elias, V. E., Ortega, J. F., Munoz, G., Munoz-Guerra, J., & Mora-Rodriguez, R. (2013). Neuromuscular responses to incremental caffeine doses: performance and side effects. *Medicine & Science in Sports & Exercise*, 45(11), 2184–2192. Doi: 10.1249/MSS.0b013e31829a6672 23669879

[ref44] Paoli, A., Cerullo, G., Bianco, A., Neri, M., Gennaro, F., Charrier, D., & Moro, T. (2024). Not Only Protein: Dietary Supplements to Optimize the Skeletal Muscle Growth Response to Resistance Training: The Current State of Knowledge. *Journal of Human Kinetics*, 91, 225–244. 10.5114/jhk/186660 38689582 PMC11057611

[ref45] Pereira, P. E., Azevedo, P., Azevedo, K., Azevedo, W., & Machado, M. (2020). Caffeine supplementation or carbohydrate mouth rinse improves performance. *International Journal of Sports Medicine*, 42(02), 147–152. 10.1055/a-1212-0742 32851636

[ref46] Pickering, C. (2019). Are caffeine’s performance-enhancing effects partially driven by its bitter taste?. *Medical Hypotheses*, 131, 109301. 10.1016/j.mehy.2019.109301 31443771

[ref47] Raya-González, J., Rendo-Urteaga, T., Domínguez, R., Castillo, D., Rodríguez-Fernández, A., & Grgic, J. (2020). Acute Effects of Caffeine Supplementation on Movement Velocity in Resistance Exercise: A Systematic Review and Meta-analysis. *Sports Medicine (Auckland, N.Z.)*, 50(4), 717–729. 10.1007/s40279-019-01211-9 31643020

[ref48] Ryan, E. J., Kim, C. H., Fickes, E. J., Williamson, M., Muller, M. D., Barkley, J. E., Gunstad, J., & Glickman, E. L. (2013). Caffeine gum and cycling performance: A timing study. *Journal of Strength and Conditioning Research*, 27, 259–264. Doi: 10.1519/JSC.0b013e3182541d03 22476164

[ref49] Santos, V., Felippe, L., Almeida Jr, J., Bertuzzi, R., Kiss, M., & Lima-Silva, A. (2014). Caffeine reduces reaction time and improves performance in simulated contest of taekwondo. *Nutrients*, 6(2), 637–649. 10.3390/nu6020637 24518826 PMC3942723

[ref50] Tarnopolsky, M., & Cupido, C. (2000). Caffeine potentiates low frequency skeletal muscle force in habitual and nonhabitual caffeine consumers. *Journal of Applied Physiology*, 89(5), 1719–1724. 10.1152/jappl.2000.89.5.1719 11053318

[ref51] Tallis, J., James, R. S., Cox, V. M., & Duncan, M. J. (2012). The effect of physiological concentrations of caffeine on the power output of maximally and submaximally stimulated mouse EDL (fast) and soleus (slow) muscle. *Journal of Applied Physiology (Bethesda, Md.: 1985)*, 112(1), 64–71. 10.1152/japplphysiol.00801.2011 21979804

[ref52] Teimouri-Korani, H., Hemmatinafar, M., Willems, M. E., Rezaei, R., & Imanian, B. (2025). Individual responses to encapsulated caffeine and caffeine chewing gum on strength and power in strength-trained males. *Journal of the International Society of Sports Nutrition*, 22(1), 2495228. 10.1080/15502783.2025.2495228 40249126 PMC12010647

[ref53] Timmins, T. D., & Saunders, D. H. (2014). Effect of caffeine ingestion on maximal voluntary contraction strength in upper-and lower-body muscle groups. *Journal of Strength & Conditioning Research*, 28(11), 3239–3244. DOI: 10.1519/JSC.0000000000000447 25144133

[ref54] Van Cutsem J., De Pauw K., Marcora S., Meeusen R., Roelands B. (2018) A caffeine-maltodextrin mouth rinse counters mental fatigue. *Psychopharmacology*, 235(4), 947–958. Doi: 10.1007/s00213-017-4809-0 29247343

[ref55] Venier, S., Grgic, J., & Mikulic, P. (2019a). Caffeinated Gel Ingestion Enhances Jump Performance, Muscle Strength, and Power in Trained Men. *Nutrients*, 11(4), 937. 10.3390/nu11040937 31027246 PMC6520843

[ref56] Venier, S., Grgic, J., & Mikulic, P. (2019b). Acute Enhancement of Jump Performance, Muscle Strength, and Power in Resistance-Trained Men After Consumption of Caffeinated Chewing Gum. *International Journal of Sports Physiology and Performance*, 14(10), 1415–1421. 10.1123/ijspp.2019-0098 30958062

[ref57] Warren, G. L., Park, N. D., Maresca, R. D., McKibans, K. I., & Millard-Stafford, M. L. (2010). Effect of caffeine ingestion on muscular strength and endurance: a meta-analysis. *Medicine and Science in Sports and Exercise*, 42(7), 1375–1387. 10.1249/MSS.0b013e3181cabbd8 20019636

[ref58] Wilk, M., Filip, A., Krzysztofik, M., Maszczyk, A., & Zajac, A. (2019). The acute effect of various doses of caffeine on power output and velocity during the bench press exercise among athletes habitually using caffeine. *Nutrients*, 11(7), 1465. Doi: 10.3390/nu11071465 31252655 PMC6682895

[ref59] Wilk, M., Krzysztofik, M., Maszczyk, A., Chycki, J., & Zajac, A. (2019). The acute effects of caffeine intake on time under tension and power generated during the bench press movement. *Journal of the International Society of Sports Nutrition*, 16, 1–7. 10.1186/s12970-019-0275-x 30777094 PMC6379960

[ref60] Wu, W., Chen, Z., Zhou, H., Wang, L., Li, X., Lv, Y., Sun, T., & Yu, L. (2024) Effects of Acute Ingestion of Caffeine Capsules on Muscle Strength and Muscle Endurance: A Systematic Review and Meta-Analysis. *Nutrients*, 16(8), 1146. 10.3390/nu16081146 38674836 PMC11054210

